# An Introductory Lecture, by Professor Lindsly, of the Columbian College

**Published:** 1840-12

**Authors:** 


AN INTRODUCTORY LECTURE, BY PROFESSOR LINDSLY, OF THE COLUMBIAN COLLEGE. *November*, 1840.

This Lecture, the subject of which is "Medical Science and the Medical Profession in Europe and the United States," is one of much more than usual interest and ability. The author of it shows himself to be a scholar, of enlarged and liberal views. We should like to see his Lecture in the hands of every student of medicine in the country. Y.

